# Local Anesthesia in Dentistry

**Published:** 1911-03-15

**Authors:** Guido Fischer

**Affiliations:** University of Greifswald, Germany


### LOCAL ANESTHESIA IN DENTISTRY.

BY DR. GUIDO FISCHER, UNIVERSITY OF GREIFSWALD, GERMANY.

### GENERAL VS. LOCAL ANESTHESIA.

Not only the ever present danger to life makes general anesthetics undesirable, but also the frequent and often disagreeable secondary symptoms, with the danger to the health. Even the simple almost harmless ether narcosis, is as far as the health is conserned, nearly the same as the chloroform narcosis. Muller mentions that ether as well as chloroform can produce a fatty metamorphosis of the inner organism. Therefore post-narcotic lung diseases after ether narcoses are of great importance being as dangerous and frequent as deaths from prolonged chloroform narcosis. We shall meet with one death (apnea) in from 3000 to 4000 ether narcoses. In dentistry local anesthetics should be the only kind used, and one should resort to general anesthetics only in exceptional cases. In operating upon the surface of the body, or in easily accessible portions of the oral cavity, local anesthesia is of prime consideration and in most cases it is safe and successful. The presence of an assistant is not needed. Anesthesia can be produced quickly, and with splendid success, if a good material be used for the injection and everything be done properly, so that that there will be no disagreeable after-effects. It is known that cocain was the poineer in the now so highly appreciated local anesthesia. This agent produced a very good anesthesia after its use had been learned, and it was employed both on the surface of the body and in the oral cavity. In the mouth, where the nature of the tissues gave many difficulties in the relief of pain, a special technique for injection had to be devised, which is now so much improved that, except in very few cases, any operation can be affected quite painlessly.

### DRAW BACKS OF COCAIN.

There is one inconvenience which still attaches to cocain, viz, its often very toxic influence on the living tissues. Falk reports, up to 1890, 176 grave intoxications from cocain, and 10 deaths; these effects are no longer to be expected since cocain is now given only in very small doses. Still, even from the smallest dose one often observes a rapid pulse accelerated and difficult respiration, nervous excitement, nausea, vomiting, convulsive spasms, dyspnea, and collapse, especially in persons with an idiosyncrasy for cocain. As an antidote, in my experience, amyl nitrate (1 to 3 drops) inhaled from a linen cloth, stands first, then comes validol (5 to 7 drops) in a little water, given internally.

### ADMIXTURE OF SUPRARENIN.

The reduction of the cocain dose was made possible to a great extent by the suprarenal extract, a drug which by its power to constrict blood-vessels causes a high rise of blood pressure. It prolongs the anesthetic effect of local anesthetics and prevents the dilution of the anesthetic agent by too rapid absorption into the general blood circulation. The danger of intoxication is thereby considerably lessened, but not abolished; for the even suprarenal extract has toxic properties though given in a relatively small dose.

### NOVOCAIN.

Novocain, discovered by Einhorn in 1905, is a powder easily soluble in water and non-irritating when injected. The mucous membrane of the mouth can tolerate this alkaloid when pure without being injured, and in fresh and bleeding wounds, caused by extraction, the pure novocain can be used with great success for post-extraction pain without detriment to the healing of the wound, while one would never dare to apply cocain, which is seven times more toxic. The solution may be boiled and sterilized without becoming decomposed, and maintains its action and sterility for a long time, unlike cocain; the addition of a small quantity of thymol still further enhancing its durability. By the use of 1.5 percent. solution a good anesthesia is produced, which is raised to its full power by the addition of the suprarenal extract. Experience shows that the best mixture is one drop of a 1:1000 suprarenin solution in each ccm. of a 1.5 percent. novocaine solution. The maximum dose of the novocain for hypodermic injection is about 0.5 gram, locally in wounds 0.5 to 1 gram.

This solution, which consists of—

| | |
|---|---|
| Novocain, | 1.5 |
| Sodium chlor., | 0.92 |
| Thymol, | 0.02 |
| Aq. dest., | 100. |

can be reduced in children and feeble persons to a 1 per cent. sometimes to a 0.5 per cent. solution, other conditions being equal. Owing to the remarkable durability of the synthetic suprarenin it is possible to dispense the drug in tubes of 1 and 2 ccm. by adding synthetic suprarenin 1:1000 to a normal dose, so that now a sterile novocain suprarenin solution is on the market. It has been proved that this normal solution remains sterile for eight weeks, and according to my belief it is the most efficacious solution for use in dental practice. The use of tablets cannot be advised, our latest experiments having shown that they are often not quite sterile.

NOVOCAIN SUPRARENIN.

Although the anesthetic power of novocain in connection with suprarenin is not greater than that of cocain, it is by no means less; besides, these drugs possess the great advantage, so very important in dental surgery, of being in this concentration at least one-half less toxic than cocain, the later frequently even in the smallest doses causing serious intoxications. The novocain-suprarenin solution does not bring about such a prônounced anemia as cocain mixed with the same quantity of suprarenin; but when the dose of suprarenin is increased from one to one and one-half drops in a solution of 1 ccm., and the injection is made in the right way, it will cause an a most disagreeable anemia. With novocain no fear need be entertained that the toxic effect will be increased by this very small increase in the admixture of suprarenal extract, as I have proved by many experiments. I therefore agree with Brown that modern local anesthesia possesses in novocain a true substitute for cocain, and therefore can wholly dispense with cocain but only if a most careful technique of injection is carried out. For the success of local anesthesia does not only depend on *what*, but also on *how* we inject, a problem which will now be more fully discussed.

### TECHNIQUE OF INFILTRATION ANESTHESIA IN THE MAXILLA.

The technique of local anesthesia by injection is determined by the course of the fifth nerve, *i. e.* its second and third branches, and on the spongiosa and cortical osseous substance in the maxillary region. On examining the extero-posterior osseous frame of the upper jaw we observe high above the roots of the molars clearly defined foramina through which the larger nerve branches and bloodvessels enter. Behind the pterygoid fossa the nervus maxillaris sends out a number of smaller nerves, the nervus alveolaris superior posterior in the alveolar process of the upper jaw running along the thin internal ridge of the tuberosity and the antrum of Highmore to the molars, and also partly to the bicuspids.

Corresponding to this innervation of the postero-exterior alveolar process we find on the palatal side a large foramen in the tuberosity of the last molars, according to the age of the individual, and in edentulous persons at about $\frac{1}{2}$ cm. before the tuberosity of the palatine alveolar process. It is this large foramen palatinum anterius through which the nervus palatinus anterior enters the interior of the hard palate, spreading its branches toward the region of the front teeth.

### ANESTHETIZING THE UPPER MOLARS.

Consequently we find in the back part of the alveolar process two characteristic nerve textures, an exterior buccal one and an interior palatal one, the places where they enter being easy to reach and of great importance in the anesthesia of the upper molars. The anterior part of the upper jaw is also governed by an anterior and exterior nerve plexus of easy access, the plexus infraorbitalis, lying in the neighborhood of the foramen infraorbitale on the interior side of the aveolar process, with the nervus alveolaris supero-anterior and the foramen incisivum with the nervus naso-palatinus lying in the palatal part. The latter ramifies in the neighborhood of the bicuspids in anastomosis with the nervus palatinus anterior. This nerve region contains the upper front teeth, and extends as far as the bicuspids. The latter lie virtually in the midst of the plexus of nerves which unites the terminal portion of the palatal branch with the anterior portion, also with the naso-palatinus.

I usually produce anesthesia of the molars by way of infiltration anesthesia of the tuberositas maxillaris. The injection is made buccally above the second molar closely beneath the reflexion of the mucous membrane, and going obliquely above and behind, the needle gradually enters the periosteum, reaching finally into the highest region of the tuberositas maxillaris (by means of the curved bayonet point of the syringe), and injecting from 1 to 2 ccm. of the solution, but not before the needle of 42 mm. length has been entirely buried in the tissue.

### ANESTHETIZING THE UPPER MOLARS.

For anesthesia of the front teeth, in most cases we inject in such a manner as to pierce into the mucous membrane at the middle of the root in front of the tooth to be injected, continue at a sharp angle to the bone, fix it here at the periosteum, and direct the needle farther to the point of the root of the tooth to be anesthetized. For one tooth we inject $\frac{1}{2}$ ccm. and for several teeth from 1 to 2 ccm. of the solution.

Palatally we pierce directly from the gum margin of the tooth to be anesthetized into the depth about parallel to the alveolar process, and inject $\frac{1}{8}$ ccm. per tooth close to the apex of the root.

### ANESTHESIA BY WAY OF THE CANINE FOSSA.

In cases of periostitis and of more serious operations (resections of the root, etc.), infiltration anesthesia should be applied in the canine fossa for the anesthesia of the nervus alverolaris superior anterior, and lingually for anesthesia of the nervus naso-palatinus. In the canine fossa the alveolar cover is very thin, and the solution there difuses very rapidly. The injection of the canine fossa is at first attended with difficulties, and it is recommended to first palpate the infraorbital ridge under which lies the foramen infraorbitale, and to compress it with the third finger of the left hand, while with the thumb of the same hand we draw the lip away from the upper jaw and then puncture close to the apex of the root of the canine in the reflexion near the muscles of the lip, and continue slanting upward and slightly backward.

When the needle (of 42 mm. length) reaches the infraorbital ridge under the compressing finger-tip, we inject about 1 to $1\frac{1}{2}$ ccm. of the solution. Palatally we do no make the puncture in the center of the papilla incisiva, as this spot is extraordinarily sensative, but we sink the needle No. 17 into the tissue about parallel to the roots to be extracted, and inject several drops as the needle progresses.

### ANESTHETIZING THE UPPER BICUSPIDS.

Bicuspids, which are innervated by the textural branches of both nerves, require particular consideration. If bicuspids exclusively are to be anesthetized, it is to be recommended to insert the needle in a horizontal and backward direction at the root of the canine, closely beneath the reflexion, and directly after the insertion to inject 1 ccm. of the solution and then gradually push the needle forward on the surface of the bone. Palatally we inject $\frac{1}{4}$ ccm. of the solution somewhere between the bicuspids in the depth of the mucous membrane, continuing toward the apex of the roof.

In single teeth and in roots of the upper jaw the method of diffusion or infiltration anesthesia is not required; instead, we inject buccally above the papilla of the tooth into the submucosa of the mucous membrane, and push the short needle forward until the periosteum is reached. The needle is always directed in such a manner that in moving forward we arrive as near as possible to the apex of the root of the tooth which is to be anesthetized. Palatally, proceeding along the root cautiously to the apex, we inject generally about one-half of the quantity which is used buccally.

### TECHNICUE OF INFILTRATION ANESTHESIA IN THE MANDIBLE.

Still more than in the upper jaw is infiltration anesthesia of importance in the mandible, and here also a distinction is made between anesthesia of the back teeth and of the front teeth. For the molars one must take into consideration the well-known mandibular anesthesia. Here we inject from $1\frac{1}{2}$ to 2 ccm. in the foramen mandibulare to anesthetize the nervus mandibularis, or alveolaris inferior, which enters here into the mandibular canal.

### ANESTHETIZING LOWER MOLARS.

With the index finger of the left hand we carefully palpate the front part of the base of the ascending branch of the mandible, where two marked bony ridges are distinguished, one anterior exterior (linea obliqua externa) and one posterior interior (linea obliqua interna). Between these, at the root of the ascending branch, we find a small depression which I would like to call fovea retro-molaris, into which the palpating fingertip sinks. Just above that lies the slightly triangular folded mucous membrane called by Braun trigonum retromolare. The edge of the finger-nail is held to the linea interna and the needle inserted along the finger-nail and near but not immediately at the bony ridge in the mucous membrane, by pushing the syringe from the canine on the other side horizontally backward on the interior surface of the part of the mandible to be anesthetized, until the needle disappears. Here the injecting solution is deposited. The place of puncture is so chosen that we raise the needle to about the height of the molar masticating surfaces in the triangular mucous membrane, in children and young persons proceeding backward while lowering the needle a little, in older patients raising the needle of 42 mm. length a little. In addition, in the buccal membrane close to the gum margin of the teeth to be anesthetized about $\frac{1}{2}$ ccm. of the solution is deposited, thus obtaining infiltration anesthesia continuous with that of the mucous membrane. After about twenty minutes in adults and from ten to fifteen minutes in children, as a rule, all teeth to and including the first bicuspid are anesthetized. Without harm, both sides of the mandible can be injected if a large number of teeth have to be extracted.

### ANESTHETIZING LOWER INCISORS.

In the region of the front teeth of the mandible the mental foramen, between the first and second bicuspids, as well as a varying number of smaller foramina which are lying next to the mental protuberance and under the root of the canine, must be considered. They lie in a slight furrow on the corticalis of the fossa mentalis, and are also traversed by nerves and vessels. The lingual corticalis of the frontal portion of the mandible is also perforated by small foramina which are occupied partly by vessels, partly by end-fibers of the nervus lingualis.

The lingullis, the branch of the fifth nerve which lies in front of the fibers of the mandibularis, enters the mandible between the canine and bicuspids in small fibers. By anesthetizing this extremity of the lingualis, the accuracy of the anesthesia in the region of the front teeth is considerably improved. We insert the needle between the bicuspid and canine in the mucous membrane downward, closely along the jaw bone, and inject immediately after the puncture $\frac{1}{4}$ccm. of the solution. Besides this, from $\frac{1}{2}$ to 1 ccm. of the solution is deposited in the fossa mentalis, pushing the needle forward medially in the depth of the reflection at the level of the root of the canine. The canine and incisors on one side become anesthetized. Owing to the strong development of the corticalis of the mandible, infiltration anesthesia occupies a special place. Only in loose roots, or in youthful jaws now and then, can anesthesia of the mucous membrane be resorted to; it is produced by injection close to the papilla. For only on the alveolar border is there sufficient spongiose bone substance to permit the solution to be further diffused. Contrary to the upper jaw, the corticalis in the mandible is strongly developed from the alveolar ridge downward, preventing any diffusion owing to the absence of available channels.

UNTOWARD AFTER-EFFECTS.

Barring a few disagreeable accidents, no serious harm to the organism has ever been reported after application of the above specified novocain-suprarenin solution, which is also used by Willinger in the great dental institute of the University of Berlin. For cases which are termed intoxications from novocain, with great probability other causes such as hysteria are to be held responsible.

### LIMITATIONS OF LOCAL ANESTHESIA.

In the very numerous and interesting clinical cases treated in the course of more than three years I have been obliged only eight times to resort to ether or ethyl bromid narcosis. With the aid of local anesthesia not only can numerous operations on the buccal mucosa, resections of the apices of roots, and cyst operations be most successfully performed, but also in some cases extended resections of the aveolar process in the maxilla, for instance in a case extending from the right second incisor to the right third molar, and in the mandible in a case extending from the left bicuspid to the right canine.

In the many cases of periostitic diseases and serious abscesses local anesthesia is indicated only if the operator is able to master infiltration anesthesia by the methods described for the upper and lower jaws.

As is well known, abscesses cause special difficulties, as local anesthesia fails owing to the impossibility of making a puncture in the abscess, not to even mention the increase of pain. Here the injection should always be made near the place of infection in healthy tissue partly by way of infiltration anesthesia and partly by injecting the mucous membrane. So a seriously periostitic upper left first bicuspid should be anesthetized by infiltration anesthesia in the foramen infraorbitale, injecting between the apices of the roots of the left first molar and second bicuspid, and also the left canine and second incisor (buccally and lingually), unless one prefers using ethyl chlorid, which is very serviceable in abscesses.

If no success can be expected from local anesthesia, as for instance in ankylosis or in obstinate and timid patients I apply the rapid ether or ethyl bromid narcosis, which is also recommended by Williger; "In patients with disease or weakness of the heart in whom we cannot make use of cocain, novocain seems to be harmless. This agent, as far as we know, can be used also in pregnant and nursing women without doing any harm. In phlegmonous processes, however, local anesthesia is not to be advised."

### INDICATIONS FOR LOCAL ANESTHESIA.

It is equably suitable for bloody as for bloodless operations when the technique and solution as indicated above are used —that is to say, in the first place, for oral surgery, but also for the relief of pain in the treatment of teeth with diseased pulps. Regarding the latter, however, we must call attention to to the fact that in these cases anesthesia offers a great disadvantage in the diagnosis and therapy, because the operator lacks the important and unerring indications offered by the pain, and may especially in treating the pulp, make mistakes which without anesthesia he would never have made.

### HYPERSENSITIVE DENTINE.

Local anesthesia is favorably indicated in the preparation for crown and bridge work, and prevents pain resulting from burring in the preparation of cavities for fillings. Local anesthesia, therefore, is of importance in hypersensitivity of the dentine, and in these cases is produced in the same manner as for surgical purposes. No other method for the producing of anesthesia of the dentine can be considered equally good. Local anesthesia by subcutaneous injection is of such great importance for the reason that the anesthetic solution is resorbed without any harm accruing to the dental pulps lying within the anesthetized field.

### CONCLUSIONS.

From all these considerations local anesthesia deserves recommendation to the pre-eminent place in dentistry. General anesthesia is to be resorted to only in very rare cases. The use of cocain must be abandoned, if for no other reason than because of its specific toxic action on the heart and central organism, especially since it can be fully replaced by novocain.

After innumerable experiments with novocain, I can recommend most heartily the 1.5 per cent. novocain-thymol solution, applied with the technique as outlined above. If the cautionary measures mentioned are observed, this solution is absolutely free from danger when it is applied properly and by a skillful hand—Abtracted from February *Cosmos.—Dental Cosmos*, Feb. 1911.

